# Assessing the fatigue resistance of NiTi instruments: A DSC‐based approach to understanding temperature effects

**DOI:** 10.1111/iej.14257

**Published:** 2025-05-23

**Authors:** Emmanuel J. N. L. Silva, Jorge N. R. Martins, Murilo Priori Alcalde, Marco A. Versiani

**Affiliations:** ^1^ School of Dentistry, Grande Rio University (UNIGRANRIO) Rio de Janeiro Brazil; ^2^ Department of Endodontics Rio de Janeiro State University Rio de Janeiro Brazil; ^3^ Deparment of Endodontics Fluminense Federal University Niterói Brazil; ^4^ Faculdade de Medicina Dentária Universidade de Lisboa Lisbon Portugal; ^5^ Grupo de Investigação Em Bioquimica e Biologia Oral, Unidade de Investigação Em Ciências Orais e Biomédicas (UICOB), Faculdade de Medicina Dentária Universidade de Lisboa Lisbon Portugal; ^6^ Centro de Estudo de Medicina Dentária Baseada na Evidência (CEMDBE) ‐ Cochrane Portugal, Faculdade de Medicina Dentária Universidade de Lisboa Lisbon Portugal; ^7^ LIBPhys‐FCT UID/FIS/04559/2013, Faculdade de Medicina Dentária Universidade de Lisboa Lisbon Portugal; ^8^ Department of Endodontics, Bauru Dental School São Paulo University Bauru Brazil; ^9^ Dental Specialty Center Brazilian Military Police Belo Horizonte Minas Gerais Brazil

**Keywords:** cyclic fatigue strength, differential scanning calorimetry, endodontics, mechanical performance, NiTi instruments, temperature

## Abstract

**Aim:**

This study aims to demonstrate that phase transformation analysis, assessed via differential scanning calorimetry (DSC), offers a more comprehensive understanding of NiTi instruments' mechanical behaviour than traditional fixed‐temperature fatigue testing by evaluating phase transformation temperatures and their impact on cyclic fatigue strength of ProTaper Universal and ProTaper Gold at 20 and 35°C.

**Methodology:**

Fifty ProTaper Universal F2 (*n* = 25) and ProTaper Gold F2 (*n* = 25) NiTi instruments were compared for geometric design (blade length, helical spirals, spiral density, spiral geometry, tip design and surface quality) and metallurgical properties including elemental analysis (energy‐dispersive X‐ray spectroscopy) and phase transformation temperatures. Then, cyclic fatigue strength was tested at room (20 ± 1°C) and body (35 ± 1°C) temperatures using a standardized artificial canal and cyclic fatigue resistance test. Time to fracture (in seconds) was recorded, and data were statistically analysed using the independent Student's *t*‐test for intergroup analysis and the paired Student's *t*‐test for intragroup analysis (*α* = 5%).

**Results:**

Both instruments had a 17 mm blade with 10 spirals (0.59 spirals/mm), similar geometry and NiTi wires with near‐equiatomic nickel‐titanium ratios, differing only in metal alloy colour. DSC analysis showed distinct phase transformation temperatures, with ProTaper Universal exhibiting an R‐phase start (Rs) at 16.2°C and finish (Rf) at −12.7°C, while ProTaper Gold had an Rs at 44.0°C and Rf at 28.6°C, though both transitions occurred gradually. Fracture time was significantly longer at 20°C than at 35°C for both instruments (*p* < 0.05), with ProTaper Gold showing a greater reduction (58%) but maintaining superior cyclic fatigue strength at both temperatures (*p* < 0.05).

**Conclusion:**

The cyclic fatigue strength of NiTi instruments depends on temperature, highlighting the limits of assessing performance at a single predefined temperature. DSC offers key insights into phase transformation, enabling a better interpretation of mechanical properties beyond mechanical testing alone. Integrating metallurgical characterization with cyclic fatigue analysis improves the evaluation of NiTi instruments, ensuring a more accurate understanding of their mechanical performance.

## INTRODUCTION

Nickel‐titanium (NiTi) instruments have improved root canal preparation efficiency and safety due to their flexibility and shape memory effect (Hülsmann et al., 2005). However, their mechanical performance remains a concern, as cyclic fatigue and torsional failure can cause instrument separation. Cyclic fatigue results from repeated tensile and compressive stress cycles in curved canals, leading to crack initiation and propagation, while torsional failure occurs when an instrument binds in the canal while the shaft continues rotating, exceeding its elastic limit (McSpadden, 2007). Therefore, improving the resistance of NiTi instruments to mechanical failure is a central focus of ongoing research, driven by efforts to refine instrument design and apply advanced heat‐treatment techniques, both of which aim to enhance durability, flexibility and overall performance under clinical conditions.

Cyclic and torsional fatigue are the two main mechanical properties used to evaluate the performance of NiTi instruments with direct clinical implications (Martins et al., 2022). The torsional resistance testing is standardized by ISO (ISO 3630‐3631, 2008) to promote comparability across different endodontic instruments. This standardization likely arises from the fact that torsional failure is strongly influenced by the instrument's geometric design. Supplementing torsional test results with an analysis of the polar moment of inertia—which describes how mass is distributed around the instrument's axis of rotation—allows for a better understanding of how specific geometric features impact mechanical performance (Zanza et al., 2021). On the other hand, there is a lack of an international standard for testing the cyclic fatigue which has led to methodological variations, such as differences in artificial canal designs, rotational speeds and environmental conditions (Martins et al., 2022). Temperature is a particularly debated factor, as NiTi instruments show distinct behaviours depending on whether tested at room (~20–25°C) or body temperature (~35–37°C). While some authors advocate testing at body temperature to better simulate clinical conditions (Dosanjh et al., 2017), this oversimplifies thermal dynamics in the root canal, since intracanal temperatures do not necessarily reach 37°C, but typically range between 31 and 33.5°C (Atmeh et al., 2024; Cunningham & Balekjian, 1980; de Hemptinne et al., 2015; Silva et al., 2025). Factors like the thermal conductivity of dentine, intermittent contact between the instrument and canal walls and the exchange of irrigants at room temperature prevent uniform heating. Additionally, NiTi instruments enter the canal at room temperature and remain inside for only a few seconds, making significant core temperature changes unlikely during clinical use. Therefore, evaluating cyclic fatigue strength at a fixed temperature may not reflect clinical conditions and oversimplifies a system where temperature variations occur dynamically. In this context, relying only on fatigue testing at fixed temperatures may overlook the changing phase transformations of NiTi alloys, which play an important role in understanding the mechanical behaviour endodontic instruments.

Understanding the clinical performance of a mechanical instrument in the root canal requires recognizing that the proportions of martensite, austenite and R‐phase at a given temperature change dynamically during use (Martins et al., 2022). Relying solely on a predefined test temperature, without considering the instrument's actual phase composition, can lead to inaccurate conclusions about its mechanical behaviour. The best way to characterize the metallurgical properties of NiTi alloys is by identifying the temperature ranges where different alloy phases remain stable during thermal cycling (heating and cooling), which can be achieved using differential scanning calorimetry (DSC) (Arias et al., 2019; Martins et al., 2022). DSC measures the heat flow during phase transitions as the sample and reference are heated or cooled at a controlled rate. As the sample undergoes thermal events, such as martensitic transformation and austenite transformation, it absorbs or releases heat, detected as a change in heat flow. The resulting data provide temperature‐dependent information about phase transition temperatures, heat capacity and phase stability, helping researchers understand the material's response to temperature variations (Arias et al., 2019). Integrating DSC analysis with mechanical testing allows for a more accurate, clinically relevant evaluation of NiTi instruments, overcoming the limitations of fixed‐temperature fatigue testing and ensuring assessments reflect the intrinsic metallurgical properties of each instrument under varying conditions.

Although the use of DSC to determine the phase transformation temperatures of NiTi alloys is well established in the literature, fatigue resistance of endodontic instruments is still commonly evaluated through tests performed at a single, fixed temperature (Martins et al., 2022). While there is ongoing debate as to whether room or body temperature better simulates clinical conditions, such an approach does not account for the complex thermo‐mechanical behaviour of NiTi alloys, whose mechanical properties are closely linked to their crystallographic phase composition. Furthermore, in studies that have attempted to combine DSC analysis with cyclic fatigue testing (Arias et al., 2018; Scott et al., 2019; Seracchiani et al., 2022), comparisons have typically been made between instruments with different geometric designs. This introduces confounding variables that limit the ability to isolate the specific contribution of phase transformation behaviour to fatigue resistance. To address this gap, the cyclic fatigue strength of two NiTi instruments that share the same geometric design—ProTaper Universal and ProTaper Gold—but are manufactured from NiTi alloys with distinct phase transformation temperatures, was tested at both room (20°C) and body (35°C) temperatures. This study also employed DSC analysis to explore phase transformation behaviour and its correlation with mechanical performance, aiming to show that such analysis provides a more complete understanding of NiTi instruments than traditional fixed‐temperature fatigue testing.

## MATERIALS AND METHODS

This manuscript complies with the Preferred Reporting Items for Laboratory studies in Endodontology (PRILE) guidelines (Figure S1) and respective checklist set forward by the International Endodontic Journal.

### Sample selection

Fifty ProTaper Universal F2 (25/.08v; *n* = 25) and ProTaper Gold F2 (25/.08v; *n* = 25) NiTi instruments (Dentsply‐Sirona), each 25 mm long, were compared for geometric design, metallurgical properties and cyclic fatigue resistance at room (20 ± 1°C) and body (35 ± 1°C) temperatures. Before testing, all instruments were inspected microscopically at 13.6× magnification with LED illumination (Opmi Pico; Carl Zeiss Surgical) for defects such as blade irregularities or unwinding. No defects were found, and all instruments were considered suitable for testing.

### Design and metallurgical characteristics

Five instruments from each group were randomly selected for stereomicroscopic evaluation (×3.4 and ×13.6 magnifications) using a dental operating microscope (Opmi Pico) with a Canon EOS 500D camera (Canon, Tokyo, Japan). The active blade length, total helical spirals and spiral density (spirals per millimetre, calculated as total spirals divided by blade length) were recorded. After stereomicroscopy, the same instruments underwent scanning electron microscopy (SEM) (S‐2400, Hitachi, Tokyo, Japan) at ×20 and ×500 magnifications to assess spiral geometry (symmetrical or asymmetrical), tip design (active or inactive) and surface irregularities from manufacturing.

Elemental analysis was performed on three instruments from each group using energy‐dispersive X‐ray spectroscopy (EDS) with a standard SEM unit (DSM‐962, Carl Zeiss Microscopy GmbH, Jena, Germany) and an Inca X‐act EDS detector (Oxford Instruments NanoAnalysis, Abingdon, United Kingdom) operated at 20 kV and 3.1 amperes after a 10‐min vacuum process. Data were collected from a 500 × 500 μm area for 1 min at a 25 mm working distance. The ZAF correction method accounted for atomic number (Z), absorption (A) and fluorescence (F) effects, ensuring accurate quantification. The corrected data were processed using specialized software (Microanalysis Suite v.4.14; Oxford Instruments NanoAnalysis, Abingdon, United Kingdom) to determine metallic element proportions.

DSC assessed phase transformation temperatures following ASTM guidelines (ASTM International, 2004). This protocol measures martensitic and austenitic transitions by tracking heat flow changes with temperature. Fragments (4–5 mm, 5–10 mg) were cut from each instrument's active blade and etched for 2 min in a solution of 45% nitric acid, 25% hydrofluoric acid and 30% distilled water. After neutralization with distilled water, specimens were placed in an aluminium pan inside the DSC device, with an empty pan as the control. The heat cycle lasted 1 h 40 min under a nitrogen atmosphere, with temperatures ranging from −150°C to 150°C at 10°C/min. DSC data and graphs were processed using Netzsch Proteus Thermal Analysis software (Netzsch‐Gerätebau GmbH, Selb, Germany). Phase transformation temperatures were estimated using the tangent method for its reproducibility in identifying transformation points (Mohajeri et al., 2020). This approach applies linear extrapolations at the inflection points of endothermic and exothermic peaks corresponding to austenitic and martensitic transformations. The intersections of these tangents with the baseline define the start and finish temperatures of each phase transformation.

### Cyclic fatigue test

**FIGURE 1:**
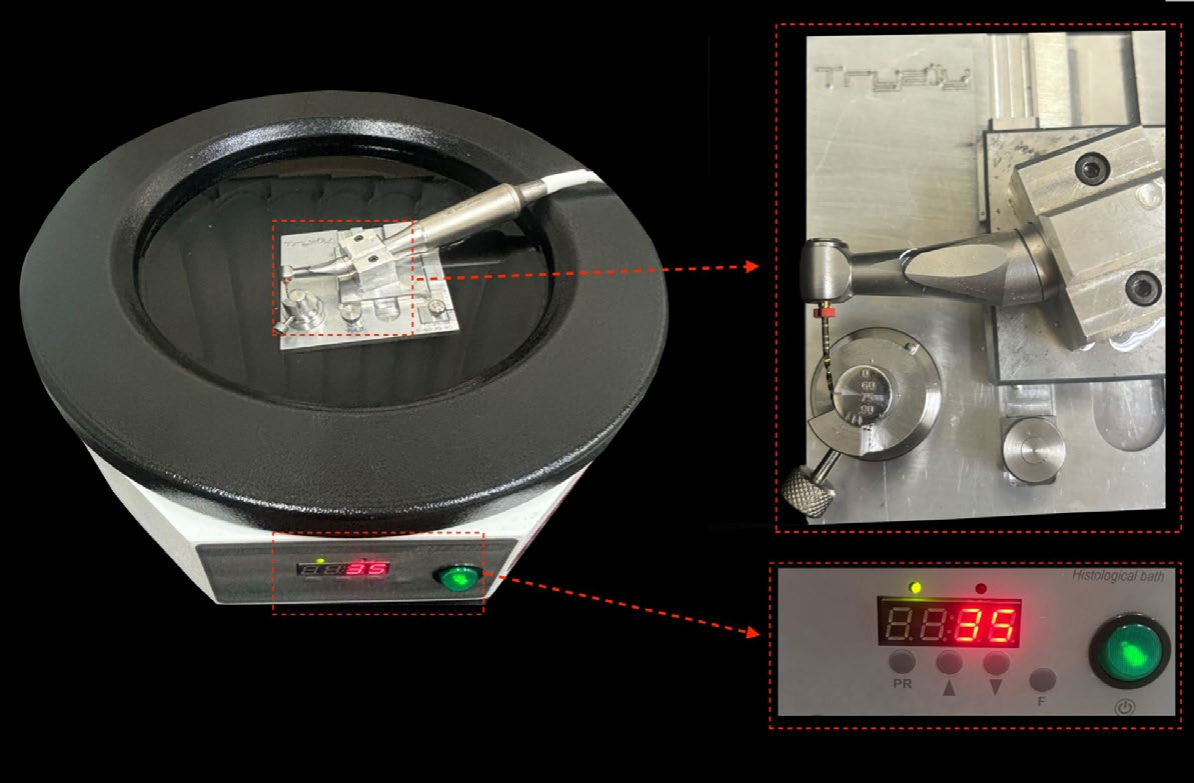
Experimental setup. For the cyclic fatigue test, the instrument was operated inside a stainless‐steel artificial canal with a 5 mm radius and 60° angle, mounted on a 6:1 reduction handpiece connected to a torque‐controlled motor. The entire assembly was submerged in a histological water bath containing distilled water maintained at a specific temperature during the test. The digital display shows the constant temperature control of the bath.

The cyclic fatigue test was conducted at room temperature (20 ± 1°C) and body temperature (35 ± 1°C). To ensure consistent conditions, the cyclic fatigue device was submerged in a histology water bath (Leica HI1210, Leica Biosystems, UK) with distilled water, and temperature was continuously monitored. Instruments were mounted on a 6:1 reduction handpiece (Sirona Dental Systems GmbH, Bensheim, Germany) powered by a torque‐controlled motor set at 350 rpm and 2.4 N·cm. They operated statically within a stainless‐steel curved tube with a 5 mm radius and 60° angle (Figure 1). Fracture was identified visually and audibly, and time to fracture (in seconds) was recorded with a digital chronometer. Scanning electron microscopy (Hitachi S‐2400, Hitachi, Tokyo, Japan) was used to examine the surface topography of the fractured instruments after the cyclic fatigue test.

### Statistical analysis

Data normality was assessed with the Shapiro–Wilk test. Since all data were normally distributed, cyclic fatigue resistance was compared using the independent Student's *t*‐test for intergroup analysis and the paired Student's *t*‐test for intragroup analysis. A 5% significance level was applied to all comparisons (SPSS v22.0 for Windows; SPSS Inc.).

## RESULTS

### Design and metallurgical characteristics

**FIGURE 2:**
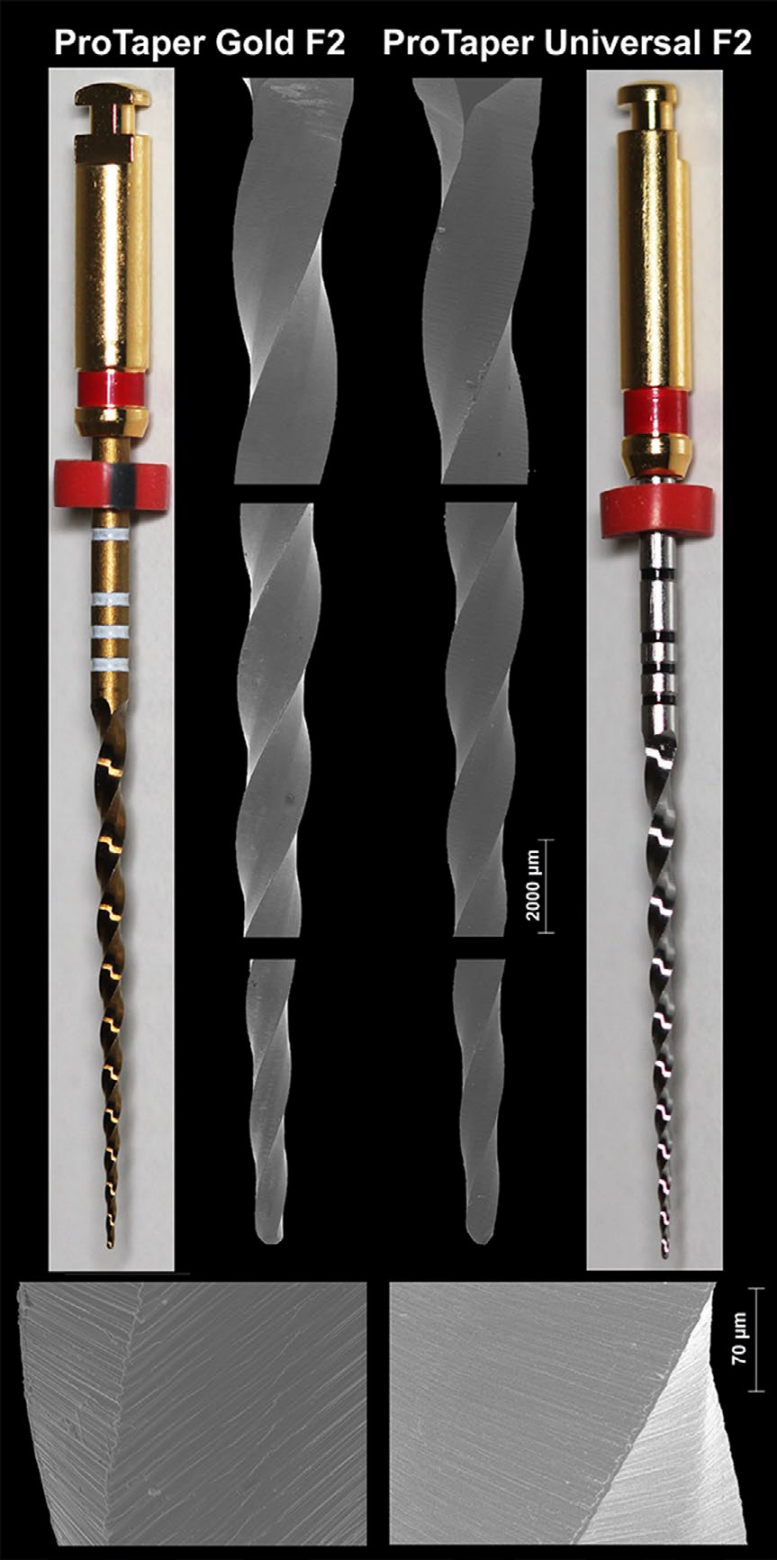
Images of ProTaper Universal (PTU) and ProTaper Gold (PTG) NiTi instruments show equivalent macro‐ and microscopic designs and surface finishes. The most evident difference is the yellowish colour of PTG, compared to the silver colour of PTU.

Both tested instruments had a 17 mm blade length with 10 spirals, resulting in a density of 0.59 spirals per millimetre. SEM inspection showed similar blade geometry, tip design and surface finish, with parallel marks from the manufacturing process. The only difference was the metal alloy colour. ProTaper Gold instruments had a yellowish colour, whereas ProTaper Universal instruments were silver (Figure 2).

**FIGURE 3:**
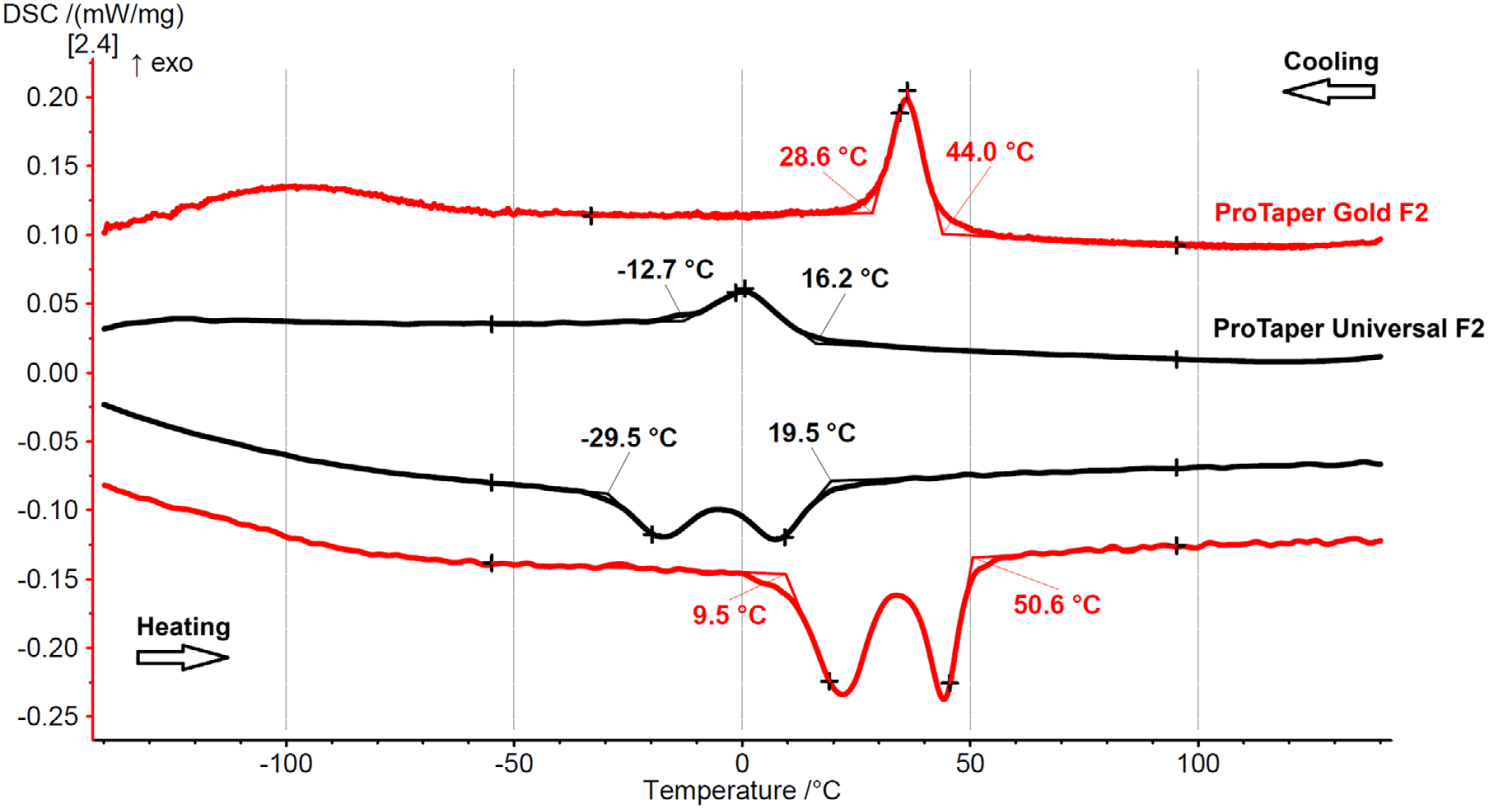
DSC charts for ProTaper Gold and ProTaper Universal instruments show cooling curves at the top (right to left) and heating curves at the bottom (left to right). Phase transformation temperature estimation was performed using the tangent method. In this approach, straight lines (tangents) are drawn along the steepest slopes of the endothermic and exothermic peaks on the DSC curve, which correspond to the austenitic and martensitic transformations. The points where these tangents intersect the baseline of the heat flow curve are used to determine the start and finish temperatures of each transformation. The phase transformation temperatures differ between the instruments. The R‐phase start (Rs) temperatures at 44.0°C for ProTaper Gold and 16.2°C for ProTaper Universal. The R‐phase finish (Rf) temperatures are estimated at 28.6 and −12.7°C, respectively. The onset of Rs occurs before while the conclusion of Rf occur after those temperatures, as indicated by the differences in the tangent line's positioning relative to the baseline.

EDS elemental analysis showed both groups consisted of NiTi wires with near‐equiatomic nickel and titanium ratios (1.054 for ProTaper Gold and 1.032 for ProTaper Universal), with no traces of other metallic elements. DSC charts revealed distinct phase transformation temperatures. ProTaper Universal showed an R‐phase start (Rs) at 16.2°C, whereas ProTaper Gold had an Rs at 44.0°C. The R‐phase finish (Rf) occurred at −12.7°C for ProTaper Universal and 28.6°C for ProTaper Gold. However, the DSC charts suggest that Rs begins and Rf ends gradually before these temperatures, as shown by the tangent line differences relative to the baseline (Figure 3).

### Cyclic fatigue

**FIGURE 4:**
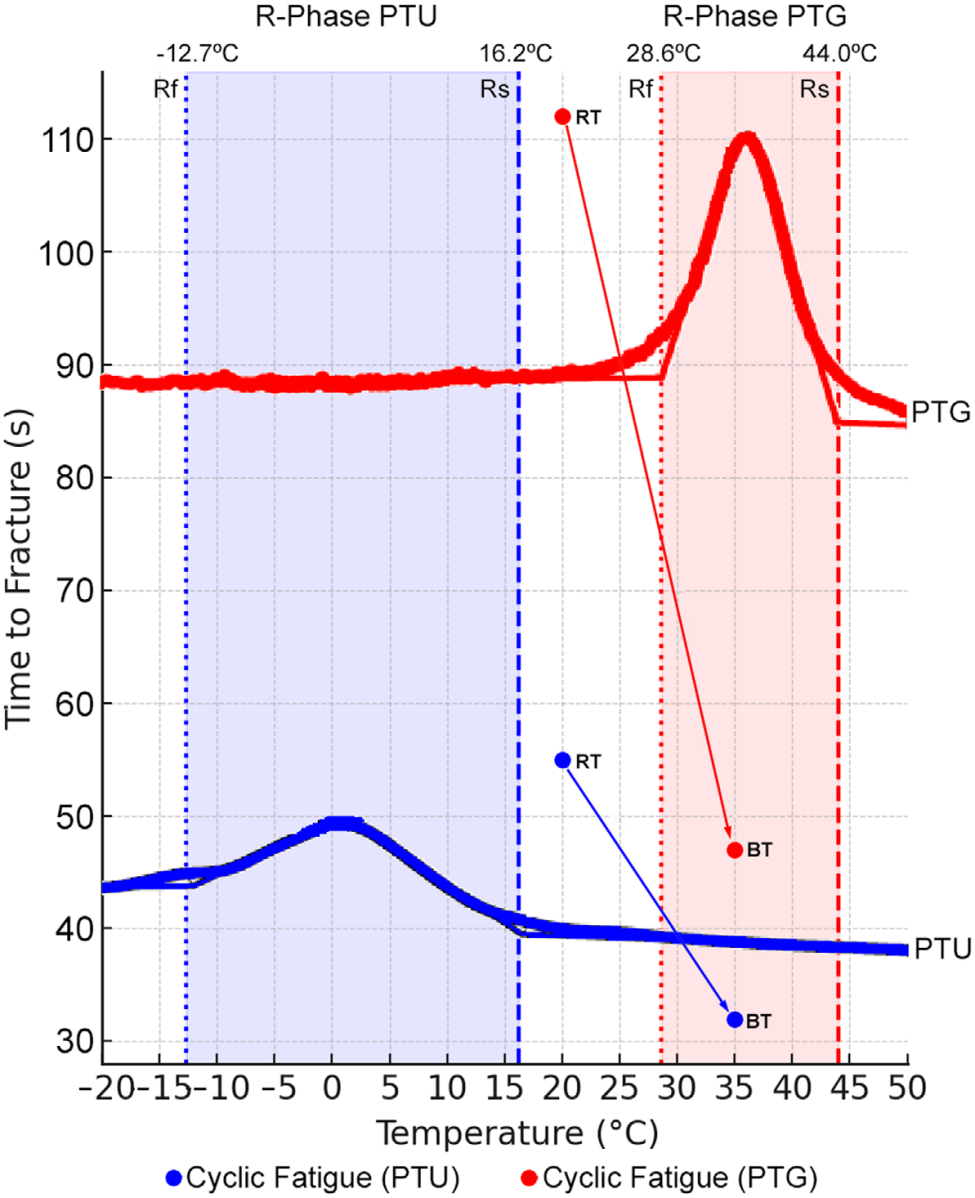
Partial view of the DSC cooling chart showing the relationship between phase transformation temperatures and cyclic fatigue outcomes. The arrows highlight that the mean fatigue strength was lower at body temperature (BT; 35°C) than at room temperature (RT; 20°C), with a more pronounced decrease in ProTaper Gold (PTG; red line) compared to ProTaper Universal (PTU; blue line). This effect results from a more significant phase transformation occurring as temperature increases within this range. Rf, R‐phase finish; Rs, R‐phase start.

**FIGURE 5:**
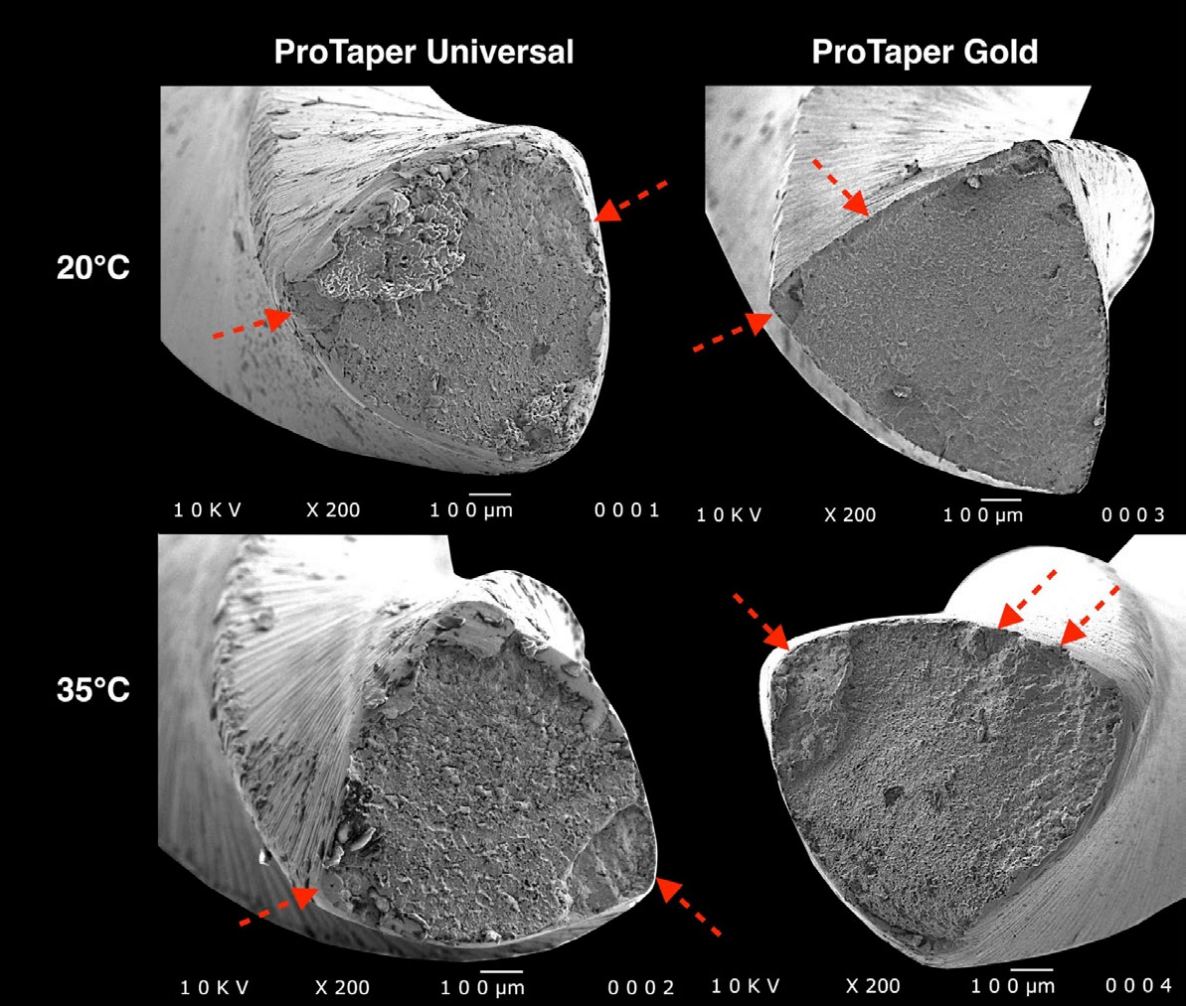
Representative scanning electron microscopy (SEM) images (×200) of the fracture surfaces of ProTaper Universal and ProTaper Gold after cyclic fatigue testing. All images exhibit characteristic features of cyclic fatigue failure, including fatigue striations and numerous dimples, indicative of ductile fracture at the final separation stage. Red arrows highlight the likely crack initiation sites, typically located near the instrument's surface, from which the crack propagated towards the centre under repeated tensile‐compressive stress cycles.

Table 1 shows the mean and standard deviation for fracture time at 20 and 35°C. Both instruments had significantly longer fracture times at 20°C than at 35°C (*p* < 0.05), indicating reduced cyclic fatigue resistance at body temperature. This reduction was greater for ProTaper Gold (58%) (Table 1, Figure 4). At both temperatures, ProTaper Gold had significantly longer fracture time than ProTaper Universal (*p* < 0.05), indicating superior cyclic fatigue strength regardless of temperature. For both instruments, the typical fractographic pattern of cyclic fatigue failure is evident, characterized by fatigue striations and numerous dimples distributed across the entire fracture surface (Figure 5).

**TABLE 1:** Mean and standard deviation of the time to fracture(s) for tested instruments at different temperatures.

	20°C	35°C	Decrease ratio (%)
ProTaper Universal	55 ± 6^Aa^	32 ± 3^Ba^	42
ProTaper Gold	112 ± 5^Ab^	47 ± 6^Bb^	58

*Note*: Uppercase letters indicate differences across temperatures for the same instrument, whereas lowercase letters represent differences between instruments at the same temperature (*p* < 0.05).

## DISCUSSION

The debate over whether cyclic fatigue testing should be conducted at room temperature or body temperature has been widely discussed, but the results of this study suggest that this debate is fundamentally flawed and oversimplified. Our findings show that fatigue performance does not depend on temperature alone, as the instrument with the highest time to fracture remained consistent across both room (20°C) and body (35°C) temperatures (Table 1). Rather, the crystallographic arrangement of the material at the tested temperature is a key factor in determining fatigue resistance, and this can be accurately assessed using DSC. This study highlights that a universal test temperature is not the ideal approach for evaluating cyclic fatigue strength. Instead, integrating DSC analysis into mechanical testing provides a more meaningful and scientifically grounded assessment of NiTi instrument performance by capturing the dynamic crystallographic changes occurring during use. Testing at predefined temperatures, such as 20°C or 35°C, may only add another experiment without offering valuable insights into the material's behaviour. DSC analysis, on the other hand, offers a precise framework for understanding phase transformation temperatures and how these affect mechanical performance.

In this study, both ProTaper Universal and ProTaper Gold exhibited significantly lower cyclic fatigue resistance at 35°C compared to 20°C (Table 1), consistent with previous research (Dosanjh et al., 2017; Keles, Eymirli, et al., 2019; Keles, Uzunoglu Ozyurek, et al., 2019; La Rosa et al., 2021; Plotino et al., 2017). This reduction has often been attributed to the decreased flexibility of austenitic NiTi, causing greater stress accumulation and faster crack propagation (Zupanc et al., 2018). However, this explanation oversimplifies the temperature–fatigue relationship. NiTi instruments exhibit varying phase transformation behaviours, and assuming a uniform response ignores the metallurgical variability among alloys and the dynamic temperature fluctuations in clinical conditions. It is important to highlight that the performance differences observed here were solely due to variations in crystallographic structure, as thorough assessments confirmed that elemental composition and geometric design were equivalent (Figure 2), ruling them out as sources of variation.

The DSC chart (Figure 3) shows that instruments with transformation temperatures close to or below body temperature remain predominantly austenitic during clinical use, exhibiting lower fatigue resistance, while those with higher transformation temperatures may retain more martensitic or R‐phase structures, potentially enhancing mechanical properties. Although this interpretation of the DSC curve provides valuable insights into the behaviour of NiTi alloy at different temperatures, a more detailed exploration of the DSC curves could yield a deeper understanding of the material's phase transformation dynamics. For example, phase transformation temperature estimation reveals that ProTaper Universal instruments maintain a fully austenitic crystallographic structure at both room and body temperatures (Figures 3 and 4). Despite this consistent arrangement, time to fracture decreased as temperature increased (Table 1, Figure 4), suggesting that factors beyond phase transformation influence fatigue resistance. While these findings align with previous research (Plotino et al., 2017), the underlying mechanisms remain unexplained in the literature, where studies have reported data without detailed analysis (Alsofi et al., 2025; Keskin et al., 2021; Teves Cordova et al., 2025; Yi Gi & Cetinkaya, 2024); however, a thorough examination of the DSC chart can clarify this behaviour.

The R‐phase transformation of ProTaper Universal begins before 16.2°C, as indicated by the positioning of the tangent line in the DSC chart (Figures 3 and 4). This suggests the true onset of transformation occurs between 30 and 40°C, coinciding with body temperature testing. Both ProTaper Gold and ProTaper Universal experience a decrease in fatigue strength due to changes in crystallographic structure (Table 1, Figure 4). However, the transformation in ProTaper Gold is more pronounced, transitioning from the R‐phase (martensitic state) at 20°C to a mixed R‐phase and austenitic state at 36°C (Figure 4), resulting in a greater reduction in fatigue strength (58% vs. 42% for ProTaper Universal) (Table 1). The DSC charts (Figures 3 and 4) clearly explain the reduction in fatigue resistance of austenitic alloys within the same austenitic phase. Although this may appear contradictory, it is substantiated by the scientific evidence presented in this study. Therefore, without DSC data, interpreting cyclic fatigue strength based on a fixed test temperature can lead to misleading conclusions, as it ignores the dynamic temperature fluctuations instruments face in clinical environments.

It is important to emphasize that the primary goal of cyclic fatigue testing is not to predict the exact clinical lifespan of an instrument but to rank instruments, designs, heat treatments or other variables based on their impact on fatigue resistance (Martins et al., 2022). Translating fracture time directly to clinical use is a misinterpretation often seen in research and clinical practice. For example, if an instrument fractures after 250 s in the test, it does not imply it can be safely used for this period of time in clinical procedures. The controlled conditions of fatigue testing do not mimic the dynamic nature of root canal instrumentation, where factors like operator technique, anatomical variations, and intermittent use affect performance (Martins et al., 2022). Thus, cyclic fatigue testing should serve as a comparative tool rather than an absolute measure of clinical durability. Instead of focusing solely on fatigue resistance at different test temperatures, a more meaningful approach is to assess the instrument's phase transformation behaviour through DSC analysis. This provides a more accurate prediction of its mechanical response in different temperature conditions, evaluating instrument performance within its true metallurgical context rather than fixed temperature benchmarks.

Although actual clinical conditions usually fall within a narrower range (~31 to 33.5°C) (Atmeh et al., 2024; Cunningham & Balekjian, 1980; de Hemptinne et al., 2015; Silva et al., 2025), the selection of 20 and 35°C was intentional, as these values are commonly reported in the literature and represent standard reference points for in vitro testing—room temperature and body temperature, respectively (Arias et al., 2019). Besides, our primary objective was to demonstrate that cyclic fatigue resistance is not determined by temperature alone but is fundamentally influenced by the crystallographic phase composition of the NiTi alloy. By choosing temperatures that span the clinical range, we aimed to highlight the importance of phase transformation behaviour in mechanical performance. Thus, intermediate temperatures were not included, as they would not have provided additional insights relevant to the aims of this study. Another important methodological consideration is that, although the temperature of the surrounding fluid is typically controlled during cyclic fatigue testing, the actual temperature experienced by the endodontic instrument may be affected by several additional factors. These include frictional heat generated between the instrument and the artificial canal walls, the instrument's geometric design and the degree of canal curvature. Such variables can lead to temperature gradients along the length of the instrument, resulting in localized regions that may reach higher temperatures than the surrounding fluid. Unfortunately, the discrepancy between the externally controlled fluid temperature and the true surface temperature of the instrument during testing remains unknown. This limitation underscores a key conclusion of the present study: that the instrument's crystallographic phase composition, rather than the nominal test temperature, plays a more decisive role in explaining the results of the cyclic fatigue resistance test.

The present findings highlight the need for an integrated approach combining mechanical testing with metallurgical characterization. Rather than standardizing fatigue testing at an arbitrary temperature, future research should focus on assessing the crystallographic arrangement of each instrument at clinically relevant temperatures (or temperature ranges) and interpreting fluctuations caused by clinical variables. For example, if an irrigating solution is used at a temperature that alters the intracanal environment, DSC analysis can help predict the instrument's mechanical behaviour under such conditions. While it does not replace mechanical testing, DSC provides valuable insights, helping researchers anticipate variations in fatigue resistance and optimise instrument knowledge. This approach enables more accurate comparisons between NiTi instruments and a deeper understanding of their real‐world performance by assessing phase dynamics within their service temperature range. Finally, while cyclic fatigue testing is particularly susceptible to temperature variations in experimental settings, temperature fluctuations will impact all mechanical performance evaluations of NiTi endodontic instruments. Thus, DSC analysis can enhance understanding of instrument behaviour not only in cyclic fatigue testing but also in other mechanical evaluations.

Future research in this area could focus on several key aspects to further understand the mechanical performance of NiTi endodontic instruments. One important direction is the development of methods to monitor real‐time temperature gradients along the instrument during cyclic fatigue testing, accounting for factors such as friction and canal curvature. This would bridge the gap between controlled fluid temperature and actual instrument temperature, providing more accurate data on phase transformation behaviour. Additionally, studying the effects of dynamic temperature protocols on fatigue resistance could better mimic in vivo conditions, allowing for a more comprehensive understanding of material behaviour under clinically relevant scenarios. Further investigation with the exploration of heat generation during clinical use may offer new perspectives on how temperature variations influence phase transitions and mechanical performance in NiTi instruments.

In conclusion, this study demonstrates that the debate over cyclic fatigue testing at room versus body temperature oversimplifies NiTi instrument performance. Our findings emphasize that crystallographic arrangement, rather than a fixed test temperature, is the key factor in explaining fatigue resistance. Integrating DSC analysis into mechanical testing enhances the understanding of phase transformation and its impact on performance, allowing for more accurate and clinically relevant evaluations by considering temperature fluctuations during clinical use. By bridging metallurgical and mechanical perspectives, this study provides valuable insights into NiTi instrument behaviour, reinforcing the importance of phase transformation analysis in future research.

## AUTHOR CONTRIBUTIONS

Emmanuel J. N. L. Silva and Jorge N. R. Martins: conceptualization, analysis, experimental procedures, writing—review and editing (lead); Murilo Priori Alcalde: experimental procedures, writing; Marco A. Versiani: conceptualization, analysis, experimental procedures, writing—review and editing (lead).

## FUNDING INFORMATION

This study was partially funded by FAPERJ and CNPq.

## CONFLICT OF INTEREST STATEMENT

The authors declare that they have no competing interests with regard to this paper.

## ETHICS STATEMENT

Not applicable.

## Supporting information


Figure S1

## Data Availability

Data that support the findings of this study are available upon request from the authors.
