# Supplementary material for: Assessing the fatigue resistance of NiTi instruments: A DSC‐based approach to understanding temperature effects
Source: Int Endod J. 2025 May 23;58(8):1267–76. doi: 10.1111/iej.14257 (PMC12254527; doi:10.1111/iej.14257)
Supplement: Supplementary file 1 — Figure S1 [file IEJ-58-1267-s001.pdf]

PRILE 2021 Flowchart

Integrating DSC analysis with mechanical testing allows for a more accurate, clinically relevant evaluation of NiTi instruments, overcoming the limitations of fixed-temperature fatigue testing and ensuring assessments reflect the intrinsic metallurgical properties of each instrument under varying conditions.

↓

This study aims to demonstrate that phase transformation analysis, assessed via differential scanning calorimetry (DSC), offers a more comprehensive understanding of NiTi instruments' mechanical behaviour than traditional fixed-temperature fatigue testing by evaluating phase transformation temperatures and their impact on cyclic fatigue strength of ProTaper Universal and ProTaper Gold at 20°C and 35°C.

↓

Ethics Committe Approval protocol: NA

↓

NiTi instruments: ProTaper Universal and ProTaper Gold

↓

ProTaper Universal (n=25), ProTaper Gold (n=25)

↓

Design (length of the active cutting blade, number of spirals, spiral geometry, spirals direction, tip design, and surface finishing), metallurgical properties (nickel and titanium composition and phase transformation temperatures), and mechanical properties (time to fracture).

↓

Scanning electron microscopy, energy-dispersive X-ray spectroscopy, differential scanning calorimetry, cyclic fatigue tests.

↓

Both instruments had a 17 mm blade with 10 spirals (0.59 spirals/mm), similar geometry, and NiTi wires with near-equiatomic nickel-titanium ratios, differing only in metal alloy colour. DSC analysis showed distinct phase transformation temperatures, with ProTaper Universal exhibiting an R-phase start (Rs) at 16.2°C and finish (Rf) at -12.7°C, while ProTaper Gold had an Rs at 44.0°C and Rf at 28.6°C, though both transitions occurred gradually. Fracture time was significantly longer at 20°C than at 35°C for both instruments ($p < 0.05$), with ProTaper Gold showing a greater reduction (58%) but maintaining superior cyclic fatigue strength at both temperatures ($p < 0.05$).

↓

DSC offers key insights into phase transformation, enabling a better interpretation of mechanical properties beyond mechanical testing alone.

↓

This study was partially funded by CAPES, FAPERJ and CNPq.

↓

The authors deny any conflicts of interest related to this study.

***From: Nagendrababu V, Murray PE, Ordinola-Zapata R, Peters OA, Rôças IN, Siqueira JF Jr, Priya E, Jayaraman J, Pulikkotil SJ, Camilleri J, Boutsioukis C, Rossi-Fedele G, Dummer PMH (2021) PRILE 2021 guidelines for reporting laboratory studies in Endodontology: a consensus-based development. *International Endodontic Journal* May 3. doi: 10.1111/iej.13542. https://onlinelibrary.wiley.com/doi/abs/10.1111/iej.13542.**

For further details visit: http://pride-endodonticguidelines.org/prile
